# Exploring fern pathosystems and immune receptors to bridge gaps in plant immunity

**DOI:** 10.1186/s12915-025-02413-6

**Published:** 2025-10-09

**Authors:** Baptiste Castel, Madeleine Baker, Jean Keller, Yves Martinez, Maxime Bonhomme, Pierre-Marc Delaux, Christophe Jacquet

**Affiliations:** 1 Laboratoire de Recherches en Sciences Végétales, CNRS, Université Toulouse III Paul Sabatier, Institut National Polytechnique Toulouse, Castanet-Tolosan, 31320 France; 2 Fédération de Recherche 3450, Plateforme Imagerie, Pôle de Biotechnologie Végétale, Castanet-Tolosan , 31320 France

**Keywords:** EvoMPMI, Fern, NLR, Filamentous pathogens, Gametophyte/sporophyte

## Abstract

Land plants include angiosperms, gymnosperms, bryophytes, lycophytes, and ferns, each of which may deploy distinct strategies to resist pathogens. Here, we investigate fern-pathogen interactions by characterizing novel pathosystems and analyzing the diversity of fern immune receptors. A collection of fern species was inoculated with a diverse set of filamentous microbes, and disease symptoms were assessed. We further leveraged published genome mining tools to analyse the diversity of receptor-like kinases, receptor-like proteins (RLKs/RLPs) and nucleotide-binding and leucine-rich repeats (NLRs), along with key immune signalling components, in ferns. Our results reveal that ferns exhibit a range of responses to pathogens, including putative non-host resistance and more specific resistance mechanisms. Among ten ferns tested, *Pteris vittata* displays the broadest spectrum of pathogen compatibility. Genome mining indicates that ferns encode a diverse repertoire of putative immune receptors, antimicrobial peptides, and mediators of systemic acquired resistance. Ferns possess numerous RLKs/RLPs, resembling those required for cell-surface immunity in angiosperms. They also encode diverse NLRs, including sub-families lost in flowering plants. These findings provide insights into disease resistance evolution and open promising perspectives for crop protection strategies.

**Supplementary Information:**

The online version contains supplementary material available at 10.1186/s12915-025-02413-6.

## Background

Ferns are an ancient plant lineage, estimated to be 400 million years old (Devonian) based on fossils and molecular data [1, 2]. But in fact, Polypodiales, which include about 80% of extant fern species, co-diversified with angiosperms around 100 million years ago, during the Cretaceous [3]. This suggests that ferns have experienced selective pressures similar to those of flowering plants. Yet their genomic evolution and environmental adaptations remain largely understudied. While angiosperm genomes have been extensively characterized, only a few fern genomes are publicly available. These include the aquatic ferns *Azolla filiculoides* [4], *Salvinia cucullata* [4], *Marsilea vestita* [5], and *Ceratopteris richardii* [6]; the terrestrial ferns *Adiantum capillus-veneris* [7] and *Adiantum nelumboides* [8]; and the tree-fern *Alsophila spinulosa* [9]. Fern genomes stand out for their large size, high chromosome numbers, long introns, and abundance of repetitive sequences.

Historically, ferns have been primarily studied through morphological traits [10, 11]. While Linnaeus originally described 11 fern genera, recent phylogenies classify the ca. 10,000 known species into 200–350 genera and 11 orders [1, 11]. Among these, Polypodiales represent 80% of all fern species, Cyatheales includes most tree ferns, Salviniales are aquatic, and Equisetales form the sister lineage to all other ferns. Ferns form a monophyletic group with spermatophytes (seed plants) with which they share vascular tissues and branched veins in the leaf, called frond in ferns. They remain distinct due to their macroscopic gametophytic phase and lack of seed reproduction [12].

Ferns can be infected by microbes, although they are overall less affected by pathogens than angiosperms [13]. In the USA alone, at least 746 filamentous pathogens have been reported on ferns, including ascomycetes such as *Colletotrichum* [14] and *Fusarium* species [15], basidiomycetes such as *Puccinia* and *Hyalopsora* species, and oomycetes such as *Pythium* and *Achlya* species [16]. These genera contain major pathogens of angiosperm crops [17, 18], suggesting that ferns could serve as a valuable genetic reservoir for disease resistance in crops.

Understanding how ferns defend themselves against pathogens could reveal both conserved and novel plant immunity mechanisms. Several models developed in angiosperms help explain the concept of incompatibility, i.e., when a pathogen cannot grow on its resistant host. One well-known mechanism is non-host resistance, a strong and durable form of resistance. It is defined as the immunity exhibited by an entire plant species against all strains or isolates of a given pathogen species [19]. This type of resistance relies on multiple layers, including physical barriers, pre-formed antimicrobial compounds, and inducible molecular defences [19]. Another model is the gene-for-gene concept, in which a single molecular factor from the pathogen is recognized by a single molecular factor in the plant, triggering immunity [20]. In angiosperms, the plant recognition factor is often a cell-surface or an intracellular immune receptor [21]. Cell-surface immune receptors are generally receptor-like kinases (RLKs) or receptor-like proteins (RLPs), recognize conserved pathogen-associated molecular patterns (PAMPs) and activate PAMP-triggered immunity (PTI) [22]. Intracellular immune receptors are generally nucleotide-binding and leucine-rich repeat (NLR), detect pathogen effectors and activate effector-triggered immunity (ETI) [23]. Three major classes of NLRs are the following: toll, interleukin1, and some *R*-genes product NLRs (TIR-NLRs); coiled-coil NLRs (CC-NLRs); and resistance to powdery mildew 8 NLRs (RPW8-NLRs). Bryophytes additionally contain lineage-specific kinase NLRs (Kin-NLRs) and α/β-hydrolase NLRs (Hyd-NLRs) [24–26]. PTI and ETI cooperatively enhance plant resistance to pathogens [27, 28].

Fern genomes encode RLKs, including families that are well described in angiosperms such as LRR-RLKs and LysM-RLKs [29, 30]. They also encode NLRs, including TIR-NLRs and RPW8-NLRs [31]. Pioneering work on NLR biology in non-flowering plants has shown that some fern NLRs retain immune-related functions [31]. Particularly, the N-terminal domain of some fern NLRs is sufficient to trigger a hypersensitive response in *Nicotiana benthamiana*, a typical proxy for testing NLR activity [31].

In this study, we investigated fern pathosystems by testing cross-compatibility between various ferns and filamentous microbes, including major crop pathogens. We also explored the diversity of immune receptors in ferns to assess their potential as a genetic reservoir for disease resistance. Our results showed that some crop pathogens, such as *Sclerotinia sclerotiorum* and *Fusarium proliferatum*, can infect multiple fern species. Among them, the fern *Pteris vittata* appears to be a particularly suitable model for studying fern immunity, as it displays various levels of resistance to a broad range of tested filamentous pathogens. Surprisingly, we found that gametophytes of *Pteris vittata* exhibit distinct resistance levels to certain pathogens compared to sporophytes. Additionally, we identified a diverse repertoire of immune receptors in ferns, including well-characterized TIR-NLRs, CC-NLRs, and RPW8-NLRs but not the bryophyte-specific Kin-NLRs and Hyd-NLRs. Furthermore, we detected also non-canonical NLRs and NLR sub-families lost in angiosperms. These results highlight the potential of ferns as a resource to explore plant immunity and improve disease resistance in crops.

## Results

### Ferns are hosts for various pathogens

**Fig. 1:**
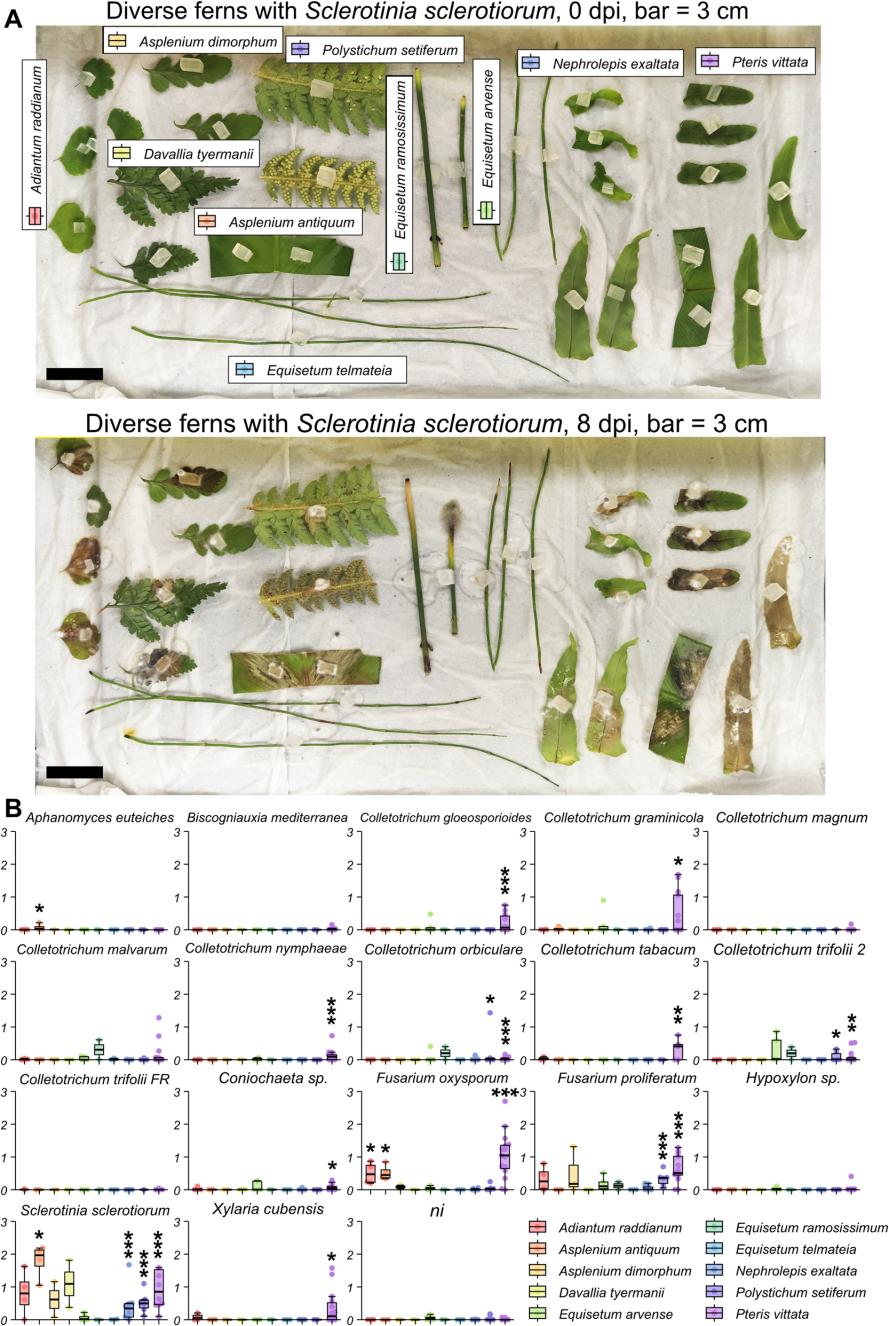
Some filamentous microbes cause disease symptoms in ferns (sporophyte). **A** Representative disease symptoms caused by *Sclerotinia sclerotiorum* on diverse ferns. Detached fronds were inoculated with plugs of PDA containing actively growing fungus. Photos were taken at 0 dpi (top panel) and 8 dpi (bottom panel). Fern species names are indicated in the top panel. Bar = 3 cm. **B** Quantification of disease symptoms caused by filamentous microbes on ferns. Fronds (or stem for *Equisetum spp.*) were collected and placed on a moist paper towel as in panel “A.” Tissues were inoculated with PDA plugs containing actively growing microbes. Disease symptoms were measured at 8 dpi, as the area (in cm^2^) of brown tissue extending from the inoculation sit, using ImageJ. Due to limited plant material, the number of replicates (*n*) ranges from 2 to 15 (average ~ 5) across independent inoculations. *, **, or *** indicate *p*-value < 0.05, < 0.01, or < 0.001 respectively, from a *t*-test for normally distributed data or a Wilcoxon test for non-normally distributed data (normality was assessed using a Shapiro test), for values with a mean > 0.05 cm^2^, as smaller values are too small to reliably describe symptoms. Figure generated with the R package “tidyplots”

We assessed the compatibility between diverse ferns and filamentous microbes (Additional file 1: Table S1 and Additional file 2: Fig.S1) [32–40]. Disease symptoms were quantified as the area of brown tissue, 8 days after inoculation using agar plugs containing filamentous microbes (Fig. 1). Several microbes did not induce visible symptoms in any of the ferns tested. These include microbes that are not pathogenic on their natural host: *Hypoxylon sp.*, *Biscogniauxia mediterranea*, *Coniochatea sp.* [36]. They also include host-specific pathogens, such as *Aphanomyces euteiches* and *Colletotrichum magnum*, suggesting non-adapted isolates or non-host resistance in ferns [19]. *Colletotrichum trifolii* race FR was also not compatible with any fern tested, but race 2 was able to grow on *Polystichum setiferum* and *Pteris vittata* (Fig. 1B). This suggests that the lack of symptoms observed for race FR is more likely due to a specific resistance mechanism in these species, rather than non-host resistance. In contrast, *Sclerotinia sclerotiorum* and *Fusarium proliferatum* induced significant disease symptoms in multiple fern species and exhibited the broadest host range among the pathogens tested. Among the ferns, *Pteris vittata* was susceptible to nearly all tested filamentous pathogens, whereas *Nephrolepis exaltata*, *Polystichum setiferum*, and the three *Equisetum* species tested displayed broad resistance. Overall, these findings indicate that some generalist filamentous pathogens, including those known to infect angiosperms, can also infect ferns. Notably, *P. vittata* appears to be broadly compatible with many filamentous pathogens.

### Gametophytes and sporophytes respond differently to some pathogens

**Fig. 2:**
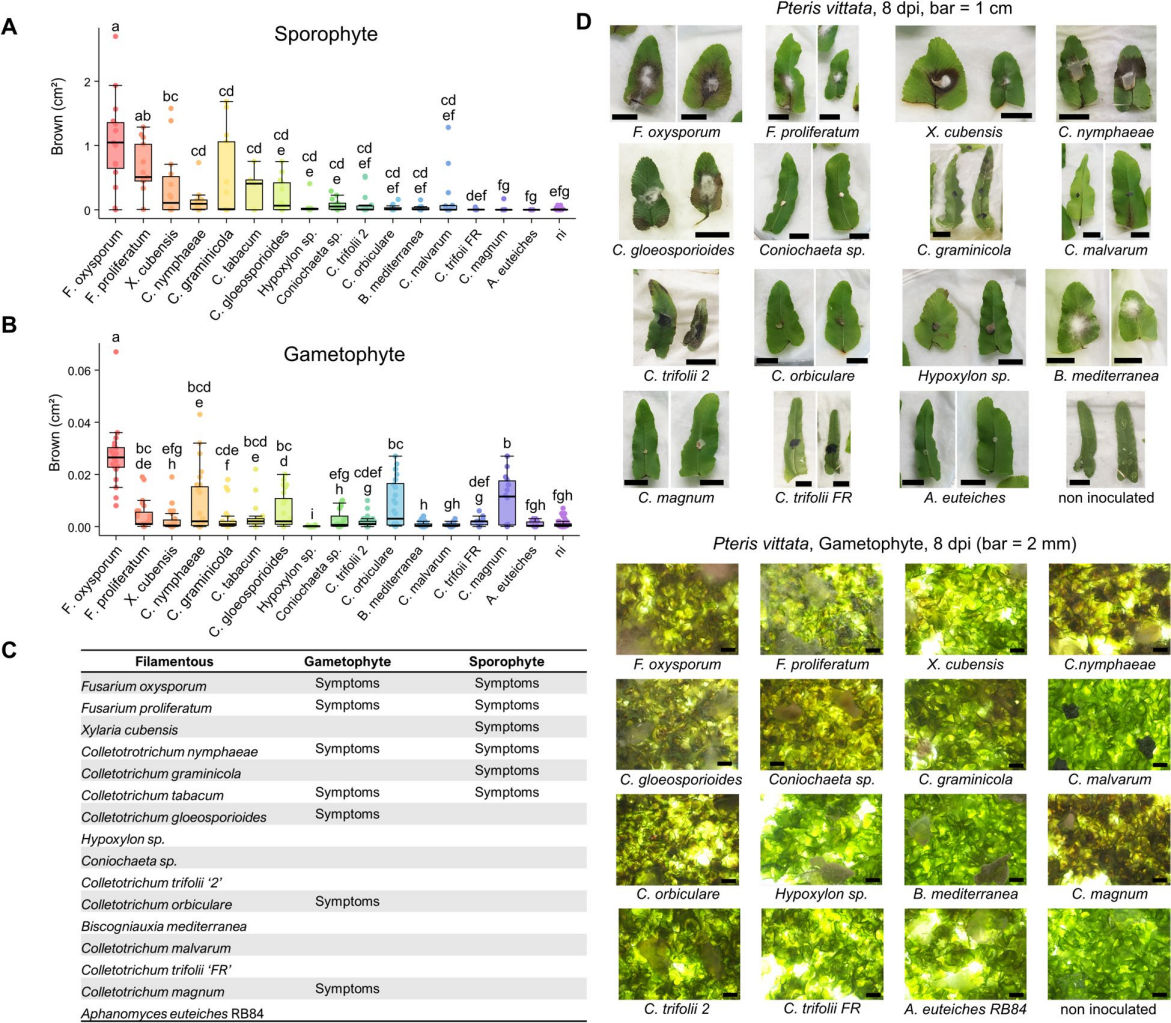
Sporophytes and gametophytes of *Pteris vittata* respond differently to some filamentous microbes. **A**, **B** Quantification of disease symptoms on sporophytes (**A**) or gametophytes (**B**) of *Pteris vittata*. Disease symptoms were measured as the area (cm^2^) of brown tissue extending from the inoculation site at 8 dpi. Letters indicate significantly different groups, analysed independently for each plot A and B, based on an ANOVA followed by a Duncan test on data normalized by quantiles, with significance threshold *p*-value < 0.05. Plots were generated with the R package “tidyplots.” **A** Detached fronds of sporophytes were inoculated with plugs containing a given microbe. Due to limited plant material, the number of replicates (*n*) ranges from 5 to 15 (average ~ 13), across independent inoculations. **B** Gametophytes of *Pteris vittata* were grown under sterile conditions and inoculated with plugs containing a given microbe. Due to limited plant material, the number of replicates (*n*) ranges from 17 to 36 (average ~ 27), across independent inoculations. **C** Table summarising significant symptoms for each pathogen, as presented in A and B. “Symptoms” indicates a significant difference with the non-inoculated plants. **D** Representative images of symptoms in *Pteris vittata* sporophytes (bar = 1 cm) and gametophytes (bar = 2 mm). Photos were taken at 8 dpi

The fern life cycle consists of two morphologically and functionally distinct phases: the haploid gametophyte, which produces gametes via mitosis, and the diploid sporophyte, which develops after fertilization and represents the dominant life stage in most species [41]. Because sporophytes and gametophytes differ in structure and physiology, particularly in the presence of vascular tissues, cuticle development, and overall size, we tested whether they could respond differently to the same pathogens (Fig. 2).

Pathogen inoculations revealed distinct susceptibility patterns in *P. vittata* sporophytes (Fig. 2A and D). Two tested *Fusarium* species were highly aggressive, inducing strong symptoms that initially localized along the veins before spreading to surrounding frond tissues. Similarly, *Xylaria cubensis*, a natural pathogen of *Marchantia polymorpha* [36], generated large necrotic and dead tissue areas, suggesting an active necrotrophic behavior. A second group of pathogens, including *C. nymphaeae*, *C. graminicola*, and C*. tabacum*, triggered maceration symptoms but in a less frequent or less extensive manner. Finally, the remaining filamentous microbes exhibited rare, weak or no visible pathogenic effects, failing to induce significant symptoms across different assays.

Gametophytes exhibited lower symptom levels compared to sporophytes, suggesting a slower progression of fungal colonization (Fig. 2B and D). Moreover, gametophytes are not infected by the same range of filamentous pathogens as sporophytes (Fig. 2 A–C) *F. oxysporum, F. proliferatum*, *C. nymphaeae*, and *C. tabacum*, caused significant symptoms in both sporophytes and gametophytes. In contrast, *C. graminicola* and *X. cubensis* caused severe symptoms in sporophytes but were unable to establish infection in gametophytes, which generally remain green. Conversely, some pathogens that failed to induce symptoms in sporophytes caused significant browning in gametophytes, such as *C. gloeosporioides*, *C. orbiculare*, and *C. magnum*.

These findings suggest that the pathogen virulence varies between gametophytes and sporophytes in *P. vittata,* highlighting a generation-dependent component of susceptibility.

### Pathogens can successfully colonize fern tissues

**Fig. 3:**
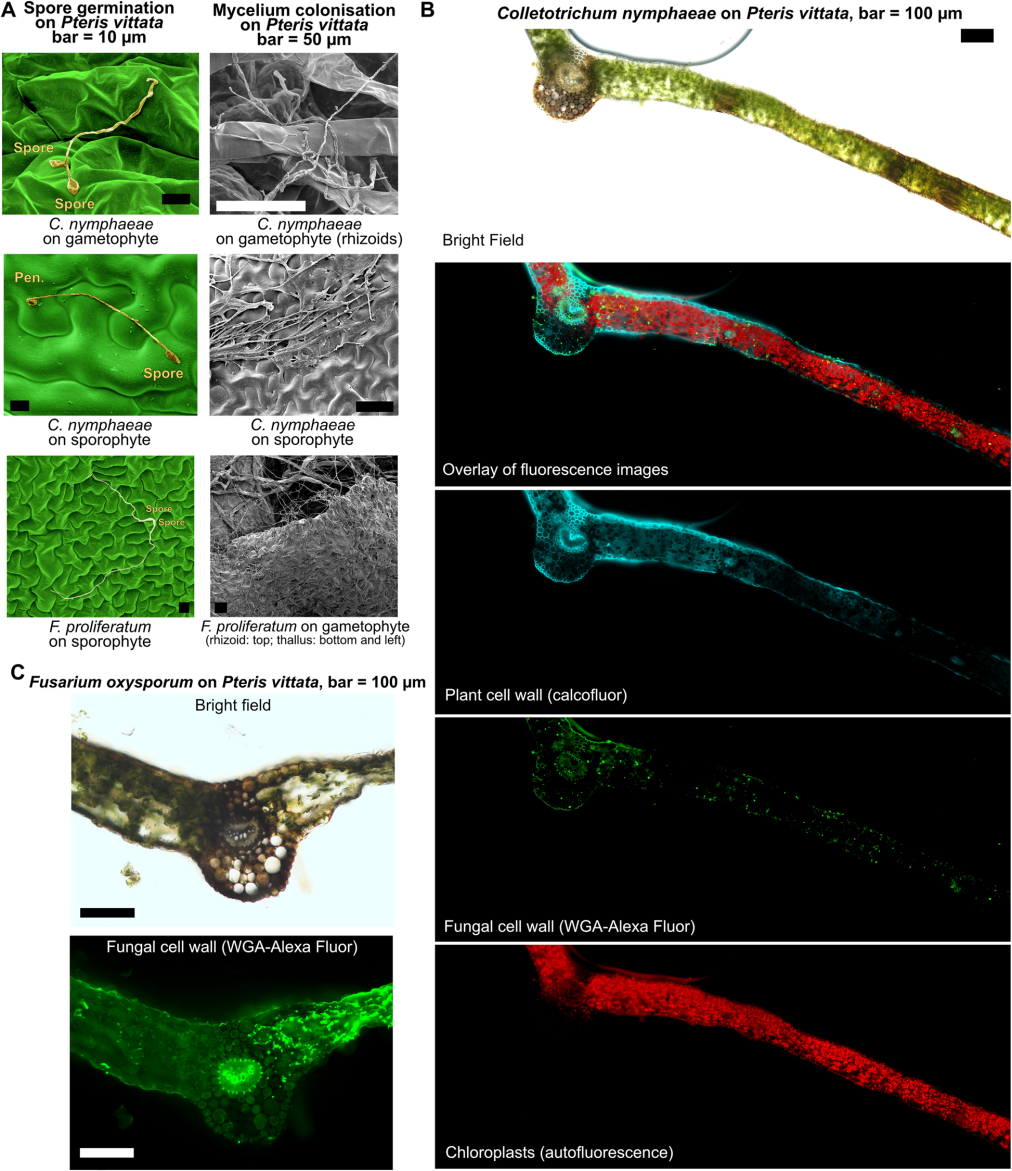
Pathogenic fungi can colonize superficial and internal tissues of *Pteris vittata. ***A** Scanning electron microscopy (SEM) images showing fungal germination and development on the epidermis of *Pteris vittata* gametophytes and fronds. Left panels: False-colored images (Affinity Designer v2.5.5), showing germinated spores and appressorium-like structures formed by *C. nymphaeae *at 2 dpi. Scale bar = 10 µm. Right panels: Later infection stages show extensive hyphal colonization of superficial tissues in *P. vittata *sporophytes or gametophytes at 6 dpi. Host cells in contact with mycelium appear swollen and macerated. Pen. = penetration structure (appressorium-like). Scale bar = 50 µm. **B** Macroscopic phenotype of *P. vittata* inoculated with spores of *C. nymphaeae*, or *F. oxysporum*. Dashed line indicates the area used for microscopy images in D. Scale bar = 1 cm. **C** Microscopy images of 100 µm cross-section *P. vittata* fronds with *F. oxysporum* showing extensive maceration and tissue disorganization on the right side of the frond, with cell wall degradation, at 4 dpi following plug inoculation. Left panel: bright field. Right panel: WGA-Alexa Fluor staining (green) highlights massive hyphal colonization in destructured tissues and intercellular fungal progression in the left-side tissue. Autofluorescence of lignin is visible in the central vasculature. Scale bar = 100 µm. **D** Microscopy images of a 100 µm cross-section of P. *vittata *frond inoculated with *C. nymphaeae*, showing a symptomatic area on the right (brown epidermis) and asymptomatic area on the left (white/transparent epidermis). Top left panel: bright field. Top right panel: calcofluor staining (cyan) shows plant cell wall degradation in the symptomatic area. Middle right panel: WGA-Alexa Fluor staining (green) shows hyphae in both symptomatic and non-symptomatic areas, with some background signal from the plant. Bottom right panel: autofluorescence (red) shows intact chloroplasts in both symptomatic and asymptomatic areas. Scale bar = 100 µm

Among the different pathogens tested, *Colletotrichum nymphaeae*, *Fusarium proliferatum*, and *Fusarium oxysporum* were selected for further microscopic analyses due to their virulence on both gametophytes and sporophytes in *Pteris vittata* (Fig. 2). We investigated their infection processes in *P. vittata* using scanning electron microscopy (SEM), confocal laser scanning microscopy, and bright-field/fluorescence microscopy (Fig. 3).

SEM images revealed that both *F. proliferatum* and *C. nymphaeae* germinated on the surface of *P. vittata* sporophyte and gametophyte tissues (Fig. 3A, left panels). *C. nymphaeae* produced appressorium-like structures, suggesting a penetration strategy similar to that on waterlilies and strawberries [42, 43]. No penetration structures were observed for *F. proliferatum*, suggesting direct penetration through cell wall digestion. At later infection stage (6 dpi), hyphae formed dense networks across the surface of both life stages. Host cells in direct contact with the mycelium appeared swollen and macerated, consistent with tissue degradation observed in necrotrophic infections (Fig. 3A, right panels). Later, fronds inoculated with droplets of conidia (13 dpi), displayed brown macerated tissues localised beneath the droplet surface. The infection appears to spread through secondary veins that darken progressively as the disease develops (Fig. 3B).

Bright-field microscopy performed in symptomatic tissues confirmed severe tissue degradation in *P. vittata* fronds caused by *F. oxysporum* (Fig. 3C), with extensive cell wall degradation and disorganized tissues, as well as massive colonization by fungal hyphae, visualized by WGA-AF fluorescence, likely indicating a switch to a necrotrophic fungal stage. *C. nymphaeae* also caused visible symptoms in *P. vittata*, characterized by browning of the epidermis (Fig. 3D, bright field panel) and of the veins, consistent with the macroscopic symptoms observed (Fig. 3B). At the microscopic level, the hyphal density appeared similar in both asymptomatic and symptomatic areas (Fig. 3D, green panel), indicating different stages of infection. Chloroplasts are visibly intact in both symptomatic and asymptomatic areas (Fig. 3D, red panel), indicating that both regions represent biotrophic phases of the fungus. However, calcofluor staining revealed a much stronger cell wall degradation in the symptomatic area (Fig. 3D, cyan panel).

Together, these findings confirm that *P. vittata* is a viable host for *C. nymphaeae*, *F. proliferatum*, and *F. oxysporum*, with successful pathogen attachment, penetration, and intercellular colonization. Many of the observed infection characteristics resemble those of plant filamentous pathogens with a hemibiotrophic lifestyle in angiosperms [44].

### Fern genomes reveal a rich diversity of putative immune receptors

Receptor-like kinases (RLKs), receptor-like proteins (RLPs), and nucleotide-binding leucine-rich repeat receptors (NLRs) are major classes of immune receptors in angiosperms [23, 45]. To determine the diversity of these receptors in ferns, we analyzed available fern genomes, along with selected reference species representing the diversity of land plants, including bryophytes (*Marchantia paleacea *[46], *Marchantia polymorpha *[47], *Physcomitrium patens *[48], *Takakia lepidozioides *[49]); lycophytes (*Diphasiastrum complanatum *[50], *Isoetes sinensis *[51], *Selaginella moellendorffii *[52]), ferns (*Alsophila spinulosa *[9], *Ceratopteris richardii *[6], *Adiantum capillus-veneris *[7], *Adiantum nelumboides *[8], *Azolla filiculoides *[4], *Salvinia cucullata *[4], *Marsilea vestita *[5]); monocots (*Dendrobium catenatum *[53], *Oryza sativa *[54], *Setaria italica *[55]); and dicots (*Camellia sinensis *[56], *Arabidopsis thaliana *[57], *Medicago truncatula *[58], *Quercus suber *[59]). We identified 11,390 RLKs (including 3,286 from ferns), 1,843 RLPs (including 371 from ferns) (Additional file1: Table S2) and 3,775 NLRs (including 396 from ferns) (Additional file1: TableS3).

#### Fern genomes encode RLKs/RLPs

**Fig. 4:**
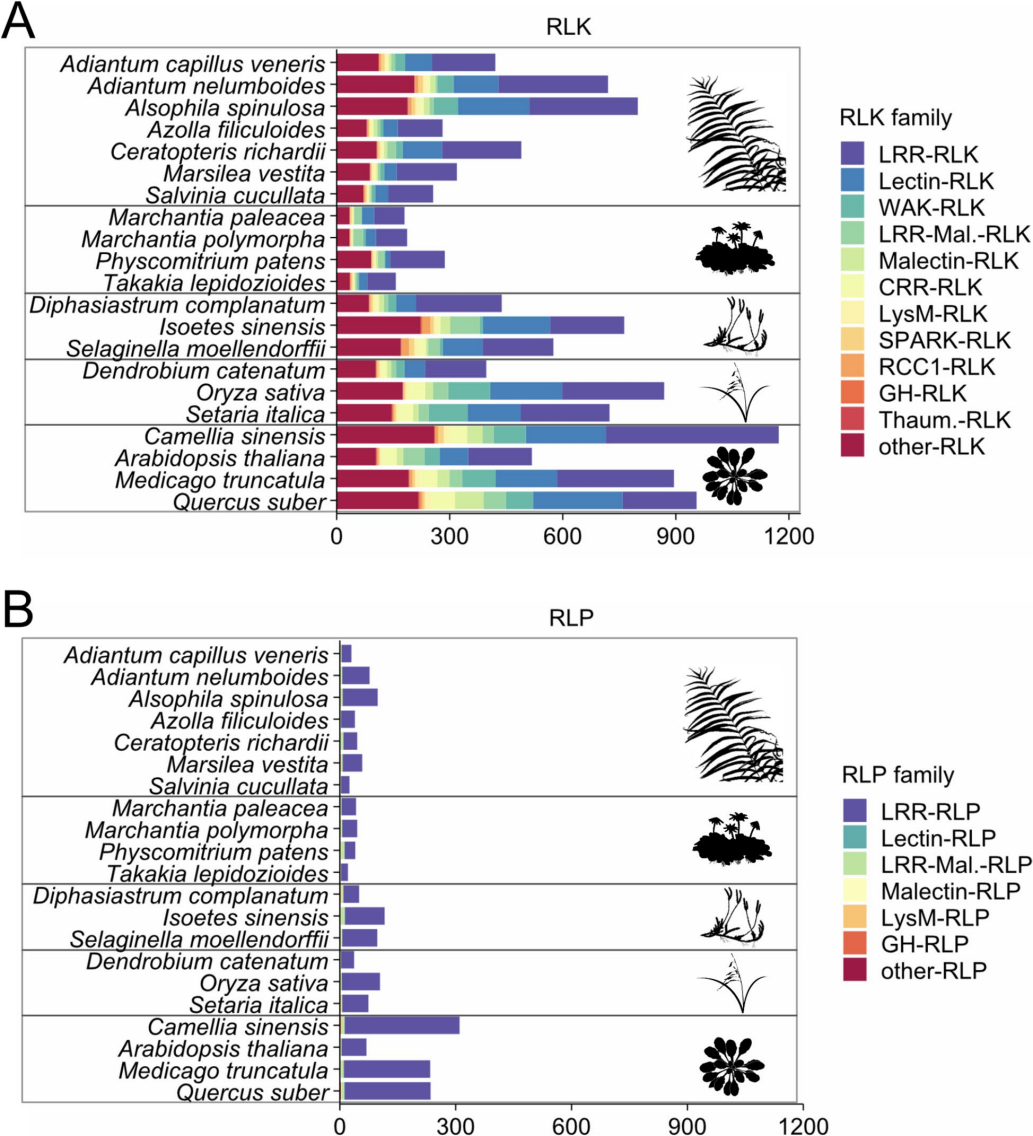
Diversity of cell-surface receptors in ferns. Distribution of receptor-like kinases (RLKs, **A**) and receptor-like proteins (RLPs, **B**) in ferns and other plant lineages. RLKs and RLPs were identified from plant genomes using RGAugury (10.1186/s12864-016-3197-x) and classified into receptor families based on HMM scan predictions. Plots were generated with the R package “tidyplots.” Cartoons representing ferns, bryophytes, lycophytes, monocots, and dicots are from PhyloPic

Within the RLK/RLP families, we identified well-known receptor classes including leucine-rich repeat (LRR), lectin, malectin, LRR-Malectin (LRR-Mal.), wall-associated kinase (WAK), thaumatin, cysteine-rich repeat (CRR), regulator of chromosome condensation 1 (RCC1), and lysin motif (LysM) (Additional file1: Table S2) [29, 60]. Their distribution in ferns is comparable to that of other land plants, with LRR- and Lectin-RLKs being the most abundant among RLKs, and LRR-RLPs being the most abundant among RLPs (Fig. 4). All categories are present in all fern genomes investigated.

In addition, some RLKs/RLPs could not be assigned to any of these categories. We investigated whether they could represent fern-specific classes. The predicted domains were mostly identical between ferns and non-ferns unclassified RLKs/RLPs (Additional file 2 : Fig. S2). We identified Romo1 (IPR018450), Vps39_1 (IPR019452), Photo_RC (PF00124), and DUF642 (PF04862) unique to ferns. However, we also identified some of these domains in tandem with kinases in angiosperms and/or gymnosperms such as A0A3P6FCZ3 (Vps39_1-kinase from *Brassica oleracea*), A0A438D9U7 (Photo_RC-kinase from *Vitis vinifera*), and A0AA38CBH3 (DUF642-kinase from *Taxus chinensis*), in the InterPro database. This indicates that these classes of proteins are not specific to ferns.

#### Fern genomes encode well-described NLRs

**Fig. 5:**
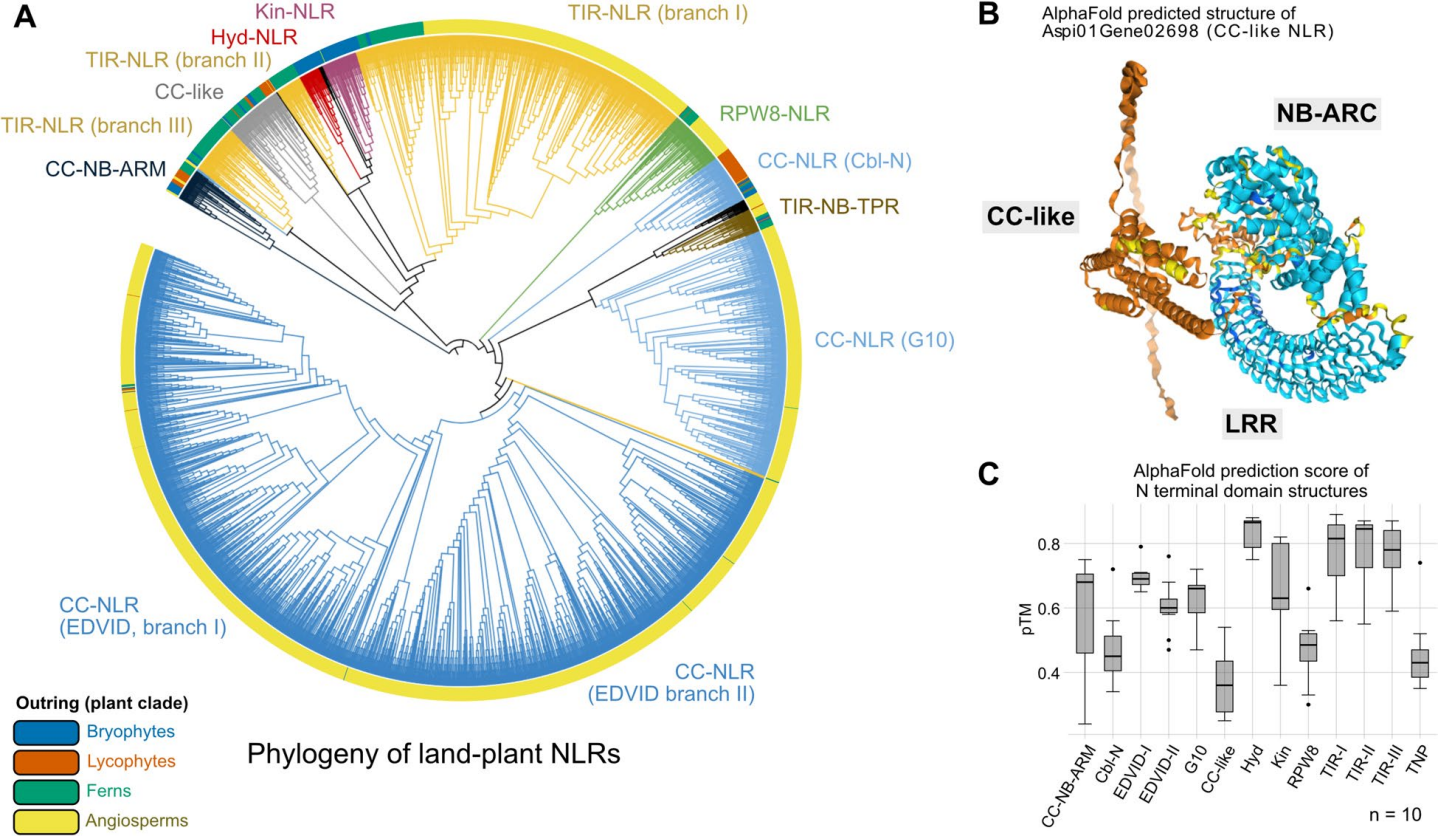
Diversity of NLRs in ferns and other land plants. **A** Phylogeny of NB-ARC domain from NLRs across representative land plants, including ferns, mosses, liverworts, lycophytes, monocots, and dicots (species detailed in Table S1). Some branches were manually colored based on annotation. The indicated clades are supported by bootstrap values > 70/100. The outer ring indicates the plant clades from which the NLR was identified. Branch lengths are not to scale. Sequences and raw tree files are available (10.5281/zenodo.17067744). **B** Structure of a representative CC-like NLR, Gene02698 from the tree fern *Alsophila spinulosa*, as predicted by AlphaFold. Dark blue indicates a very high confidence (> 90%), pale blue indicates a good confidence (70–90%), yellow indicates a low confidence (50–70%), and orange indicates a very low confidence (< 50%). **C** Plot of the predicted template modelling (pTM) scores of diverse N-terminal domains from each clade of NLRs from the phylogeny in “A.” Regions from the first amino acid to the start of the NB-ARC domain were predicted with AlphaFold for 10 representative NLRs of each clade. The structures and NLR names are presented in Figure S3

We classified NLRs using a phylogeny of their NB-ARC (Fig. 5A). Using protein domain annotations, we identified clusters of NLRs associated with known NLR families, including TIR-NLRs, CC-NLRs, and RPW8-NLRs (Additional file1: Table S3). TIR-NLRs form three distinct clusters, with “Cluster I” being the most widespread across land plants, including angiosperms, bryophytes, and ferns. “Cluster II” is specific to ferns, while “Cluster III” includes both ferns and bryophytes and is unexpectedly related to certain CC-NLRs from tea (*Camellia sinensis*), suggesting potential functional diversification. RPW8-NLRs are present across all land plant lineages. CC-NLRs segregate into three main groups: “EDVID” and “G10,” both specific to angiosperms, and “Cbl-N,” which is found only in non-angiosperms, including ferns, lycophytes, and bryophytes. These findings suggest that CC-NLRs underwent lineage-specific expansions, with “EDVID” and “G10” evolving predominantly in spermatophytes, while “Cbl-N” represents the ancestral state retained in non-angiosperms.

#### Fern genomes encode CC-like NLRs

We identified three additional clusters of fern NB-ARC-containing proteins: TIR-NB-TPRs, CC-NB-ARMs, and one unclassified lineage (Fig. 5A). TIR-NB-TPRs form a conserved class of NLRs, potentially regulating immunity, containing a TIR domain but signalling independently of TIR-NLRs [61]. CC-NB-ARMs are also conserved in plant genomes [62, 63]. The rice CC-NB-ARM “RLS1” could be a regulator of immunity [64].

The unidentified NLR branch (in grey in Fig. 5A) is closely related to Hyd-, Kin-, and TIR-NLR clusters I and II. This cluster is particularly rich in fern NLRs (Fig. 5A), prompting further investigation. In a previous study, NLRs from this cluster were referred to as “OG5” and “OG11” based on sequence, and “AF-6 (CC-like)” based on predicted structure [31]. We used AlphaFold [65] to investigate further the structure of these CC-like NLRs (Fig. 5C,D and Additional file2 : Fig.S3). Gene02698, a representative CC-like NLR from *Alsophila spinulosa*, has typical LRR, NB-ARC, and CC dmains, with a peculiar, disordered strand at the N-terminus of the CC (Fig. 5C). The LRR and NB-ARC are predicted with high confidence, while the N-terminal domain is predicted with a low confidence (Fig. 5C). In fact, the N-terminal domains of many CC-like NLRs are predicted with very low confidence, and sometimes with an N-terminal disordered region, unlike other CC domains (Fig. 5D and Additional file2: Figure S3). The actual function of CC-like NLRs requires further investigation. Notably, two CC-like domains (from Mp1g20580 and Mp4g08790 of *Marchantia polymorpha*) failed to induce a hypersensitive response in *Nicotiana benthamiana*, a typical readout of NLR activity [31].

### Known immune-related genes are conserved in fern transcriptomes

**Fig. 6:**
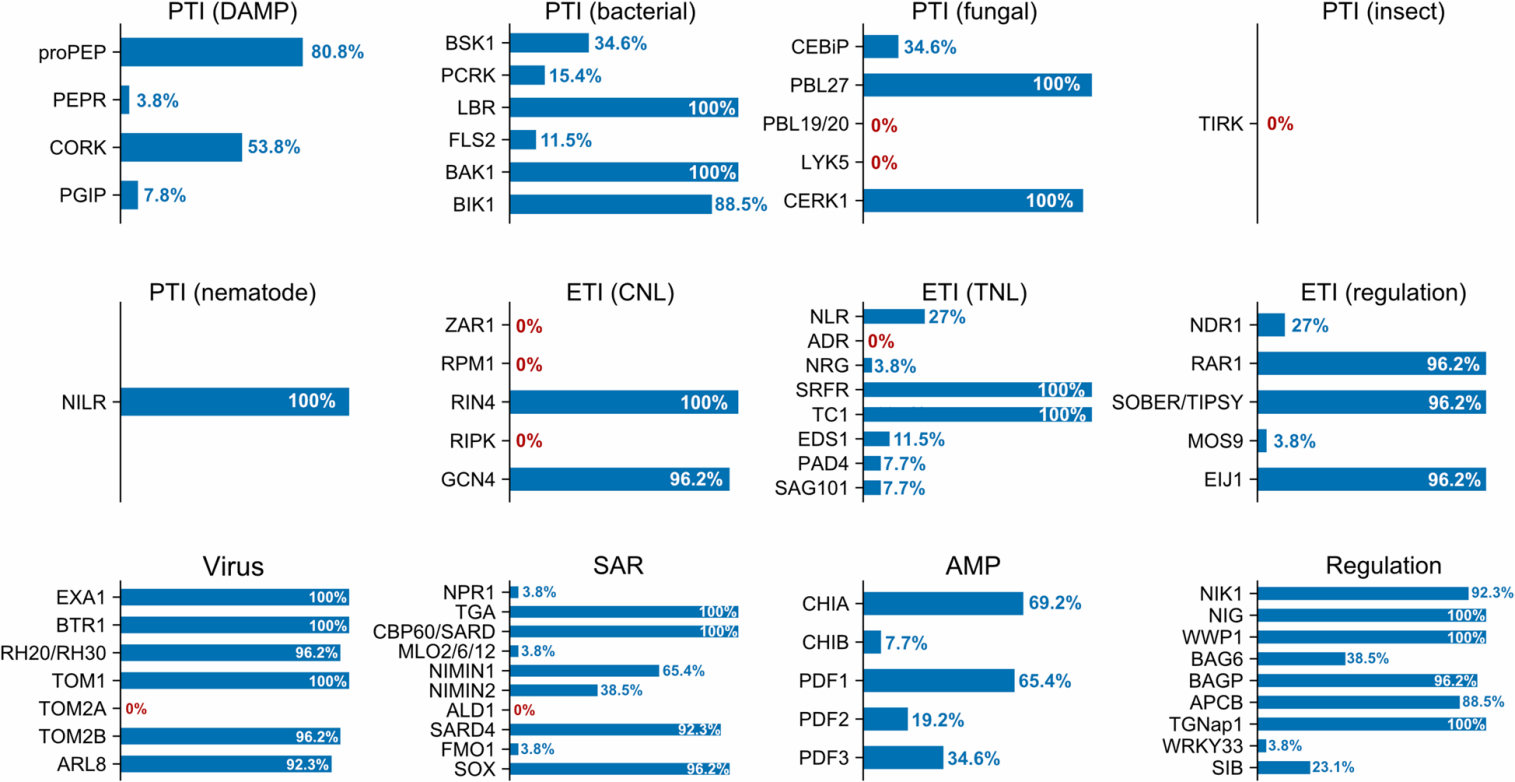
Prevalence of known immune-related genes across 26 ferns transcriptomes. Data extracted from (Ali et al., 2025, Table S9). Genes annotated as involved in response to pathogen or virus were included. The plot was made with the R package ggplot2, based on data show in Additional file 6: Table S4. Gene presence is expressed as a percentage of ferns transcriptome containing an ortholog, out of 26 transcriptomes analyzed. PTI: PAMP-triggered immunity, DAMP: Danger-associated molecular pattern, ETI: effector-triggered immunity, CNL: CC-NLR, TNL: TIR-NLR, SAR: systemic acquired resistance, AMP: antimicrobial peptide

A recent study that interrogated 26 fern genomes or transcriptomes [2] found that many immune genes are conserved in ferns (Fig. 6 and Additional file1 : Table S4). This includes *BAK1* and *BIK1* required for signalling of many LRR-RKs [22]. Two well-characterized CC-NLRs were not detected in ferns (Fig. 6). However, our phylogenetic analyses confirmed the presence in ferns of CC-NLRs (CNLs), but only from the “Cbl-N” type, which was lost in angiosperms (Fig. 5). Despite their sequence divergence, “Cbl-N” CC-NLRs likely function similarly to “EDVID” CC-NLRs found in angiosperms [31]. In addition, RIN4, a protein targeted by multiple effectors and guarded by several CC-NLRs in angiosperms [66], is well conserved in ferns (Fig. 6).

Proteins acting downstream of TIR-NLRs, such as EPs (EDS1, PAD4) and the RPW8-NLRs NRG1 and ADR1, are present in some ferns but absent in many species (Fig. 6). However, ferns encode other types of RWP8-NLRs (Fig. 5) [67].

In Arabidopsis, pathogen detection triggers the synthesis of salicylic acid (SA) that activates both local and systemic immune responses, known as systemic acquired resistance (SAR) [68]. SARD1 and CBP60g that regulate the expression of genes for SA biosynthesis such as *ICS1* and *EDS5* [69] are strongly conserved in ferns (Fig. 6). However, genes required for SA biosynthesis were not analyzed by [2]. NPR1, an SA receptor required for SAR in Arabidopsis [70], is mostly absent from ferns (Fig. 6) [50].

Additionally, antimicrobial peptides (AMP), including chitinases and defensins, are widely present in ferns (Fig. 6). This supports the idea that AMPs constitute a conserved defence strategy across eukaryotes [71–73].

## Discussion

### Ferns are susceptible to a broad range of pathogens

The literature on fern diseases is sparser than that for angiosperms [13]. Yet, several pathogens have been reported on ferns, primarily filamentous fungi [16, 74–76], but also bacteria [77–79] and viruses [80]. Some of the reported filamentous pathogens belong to the same species as crop pathogens, such as *Colletotrichum gloeosporioides* [14] and *Botrytis cinerea* [16], both causing diseases on many crops including soybean, wheat, and tomato [81, 82].

Here, we expand this category by identifying *Fusarium oxysporum*, *Fusarium proliferatum*, *Sclerotinia sclerotiorum*, *Colletotrichum nymphaeae*, and a specific race of *Colletotrichum trifolii* as pathogens capable of colonizing ferns (Fig. 1). This raises the question of whether these interactions reflect true compatibility or opportunistic infections facilitated by stress conditions in experimental settings. Microscopy observations revealed several conserved features of fungal pathogenicity (Fig. 3). Pathogens such as *C. nymphaeae* and *F. oxysporum* retained classical infection strategies, including the formation of appressorium-like structures, intercellular hyphal development, vein colonization, and an alternation between biotrophic and necrotrophic phases. These observations align with previous findings on the evolutionary dynamics of plant-parasite interactions, which suggest that many core mechanisms of pathogen virulence remain conserved despite host diversification [83].

All three *Equisetum* species tested were largely resistant to all filamentous pathogens tested (Fig. 1B). Because leaves are reduced to scale-like microphylls in horsetails, we inoculated stems or branches instead. Although horsetail stems and branches contain stomata and have taken over the photosynthetic function of the leaf [84], they remain structurally different from true leaves, which could hinder pathogen infection in our experiment. For example, they have a distinct cuticle wax layer, known to contribute to resilience against abiotic stress [85], which could also play a role in disease resistance. Horsetail can also produce antimicrobial compounds [86], which could further explain their resistance to filamentous pathogens in our experiments (Fig. 1B).

The inability of *Aphanomyces euteiches* to infect any of the tested fern species suggests non-host resistance, a widely observed phenomenon in plant-pathogen interactions where an entire plant lineage remains incompatible with a given pathogen [19]. Conversely, the differential susceptibility observed between *P. vittata* and the two tested races of *C. trifolii* strongly suggests host-race specific immunity, which may be linked to the recognition of an effector present in race FR, but not in race 2.

The ferns pathosystems reported here provide valuable insights into the evolution of plant–pathogen interactions, offering new perspectives on resistance mechanisms in land plants. Together, they also highlight the need to expand such studies to include obligate biotrophs, especially rust fungi (*Pucciniales*), which are the most frequently reported pathogens on ferns [13].

### Pteris vittata could be a model to study fern-pathogen interactions

*Pteris vittata* exhibits the broadest range of compatibility with filamentous pathogens, including those that typically infect angiosperms or bryophytes (Figs. 1 and 2). This fern is particularly interesting as it can accumulate arsenic [32, 87], likely through the arsenate reductase PvACR2 [88], and it can establish arbuscular mycorrhizal symbiosis with the fungus *Glomus intraradices* [89], making it a valuable model to investigate the conservation of the common symbiosis signalling pathway (CSP) in ferns [90]. In addition to its role in soil arsenic decontamination and symbiosis, we found that *Pteris vittata* is partially compatible with multiple pathogens, including *F. oxysporum*, *F. proliferatum*, *X. cubensis*, *C. nymphaeae*, and *C. trifolii* race 2. These findings strengthen the potential of *P. vittata* as a model to study fern-microbe interactions and the evolution of molecular plant–microbe interactions.

### Gametophyte and sporophytes are differentially resistant to pathogens

The life cycle of ferns consists of two independent and macroscopic phases: the gametophyte and the sporophyte [11]. Fern gametophytes, called prothalli, develop from spore germination. They are generally heart-shaped, one cell layer thick, and only a few millimetres in size. They generate gametes, whose fertilization gives rise to a sporophyte, the dominant form of most ferns. The sporophyte forms roots, a stem (called rhizome, even when above ground) and leaves (called fronds) which bear spore-producing sporangia. Spore germination results in a new gametophyte. These structural and physiological differences suggest that sporophytes might be more resilient to stress. Surprisingly, our results revealed that *P. vittata* gametophytes can exhibit greater resistance to some fungi such as *Colletotrichum graminicola* and *Xylaria cubensis*, challenging the assumption that gametophytes are inherently more susceptible to pathogens (Fig. 2).

Despite their seemingly fragile structure, gametophytes demonstrate remarkable resilience to environmental stresses. For instance, fern gametophytes exhibit a high tolerance to desiccation [91], a trait that is rare in sporophytes and likely a homoplasy [92, 93]. Fern gametophytes also show adaptation to light stress [94], likely through the accumulation of reactive oxygen species-scavenging molecules, such as β-carotene or tocopherols [95, 96]. Additionally, some fern gametophytes are more resistant to freezing than their sporophytes, enabling their survival under snow during winter [97]. These differences suggest that gametophytes and sporophytes deploy distinct physiological or biochemical defence mechanisms. In angiosperms, developmental stage-dependent resistance to pathogens has been also reported, with mature plants generally more resistant than seedlings [98, 99]. In the case of *P. vittata*, gametophytes appear more resistant than sporophytes to *C. graminicola* and *X. cubensis* (Fig. 2). One possible explanation is the absence of vasculature in gametophytes, which may limit systemic pathogen spread. Another possibility is the accumulation of antimicrobial compounds specific to gametophytes. Indeed, ferns are known to produce a wide range of specialised metabolites in response to pests [100–103]. Further investigation, particularly at the molecular level, is needed to determine whether gametophyte-specific immune strategies exist and how they compare to those of sporophytes.

### Putative immune receptors are conserved within tracheophytes

Tracheophytes have been exposed to pathogen infections for at least 407 million years [104] and likely since terrestrialization [26, 105, 106]. Our findings indicate that ferns can resist angiosperm pathogens, raising the question of whether these defence mechanisms are ancestral traits shared with seed plants or represent lineage-specific innovations. To investigate this question, we examined the conservation of well-characterized immune-related genes in ferns (Fig. 6).

CC-NLR-mediated immunity is ancestral to land plants, but angiosperms evolved distinct “EDVID” and “G10” classes of CC-NLRs, while other lineages including ferns retain the “Cbl-N” ancestral class (Fig. 5A). Ferns and others non-flowering plants contain NLRs with a CC-like domain, predicted with low confidence and often with a disordered N-terminal tail, or belonging to the “Cbl-N” type (Figs. 5 and Additional file 2: Fig. S3). CC-like and Cbl-N represent the ancestral state, lost in angiosperms, which in contrast have evolved distinct “EDVID” and “G10” classes of CC-NLRs.

Ferns encode numerous TIR-NLRs (Fig. 5A). Based on studies in angiosperms, TIR-NLRs require EDS1, PAD4, SAG101 (coreceptors of the TIR-NLR enzymatic product), and ADR1 and NRG1 (RPW8-NLRs recruited downstream of EDS1, PAD4 and SAG101) [107]. These proteins are absent from many fern transcriptomes (Fig. 6) [2]. A simple explanation is that these ferns evolved receptors different from EDS1, PAD4, and SAG101, specifically for fern TIR-NLR enzymatic product. Even if most ferns lack ADR1 or NRG1 (Fig. 6), they contain other types of RPW8-NLRs (Fig. 5A) [67], which could be recruited downstream of these unknown receptors. Although rare, some ferns contain close homologues of EDS1, PAD4 and SAG101 (Fig. 6) [2], indicating that this pathway is ancestral to tracheophytes, but has been lost in many ferns.

Many ferns lack the salicylic acid (SA) receptor NPR1. In *Marchantia polymorpha*, NPR1 is conserved, can complement Arabidopsis *npr1* mutants, and can bind SA, suggesting that this function is ancestral to land plants [108]. The absence of *NPR1* in many ferns raises questions about the role of SA in SARD1/CBP60g-mediated immunity and SAR in ferns, and whether alternative mechanisms exist. Pipecolic acid is another molecule required for SAR, and its biosynthesis requires SARD4 [109]. Unlike *NPR1*, *SARD4* is conserved in ferns, suggesting that pipecolic acid may play a predominant role in fern SAR.

Overall, many components of PTI and ETI appear to be ancestral to tracheophytes. However, some pathways, such as the EP-mediated regulation of TIR-NLR signalling, are reduced in ferns, suggesting the presence of yet-to-be-identified compensatory mechanisms.

### Ferns can be a reservoir of R-genes

The plant immune system relies on RLK/RLP cell-surface receptors for PTI and NLR cytoplasmic receptors for ETI [21, 27, 110]. Many disease resistance strategies in crops involve the introgression of such immune receptors [111]. Since we found that ferns can resist pathogens (Figs. 1 and 2), we propose that they could serve as natural reservoirs of immune receptors, to be transferred into crops.

We found that PTI components are largely conserved across land plants (Figs. 4 and 6). This is corroborated by functional studies showing conservation of central PTI regulators such as LysM receptors [112, 113], SERK receptors [114], CPK28 [115], or RBOH [116]. This conservation indicates that RLKs/RLPs could be prioritized as candidate determinants for resistance to pathogens in ferns.

In addition, NLRs have evolved differently across plant lineages (Fig. 5). In ferns, we found three major classes of NLRs: CC-, TIR-, and RPW8-NLRs (Fig. 5). Fern CC-NLRs belong to the “Cbl-N” cluster (Fig. 5A), which is found in bryophytes, lycophytes, and ferns, but was lost in angiosperms, suggesting that “Cbl-N” represents the ancestral state of CC-NLRs. Instead, angiosperms evolved “G10” and “EDVID” CC-NLRs. “G10” and “EDVID” expanded (Fig. 5) to become overrepresented in asterids and monocots [25]. Their success in asterids could be explained by the robustness of the “NRC” networks, formed between many CC-NLRs “EDVID” [105, 117–119]. TIR-NLRs and RPW8-NLRs are found in all lineages (Additional file1:Table S3) [67]. They have been shown to function in the same immune signalling pathway, along with the protein EDS1 [107, 120, 121]. This broad conservation suggests that it dates back to the earliest land plants. These data suggest that fern NLRs could be functional in angiosperm crops, despite millions of years of independent evolution. However, no fern NLR has yet been demonstrated to confer resistance to a pathogen.

## Conclusions

We identified novel pathosystems in ferns, some of which are likely controlled by specific resistance mechanisms. We also found that fern genomes encode a remarkable diversity of putative immune receptors, including some absent in angiosperms. These findings suggest that ferns could serve as a reservoir of immune receptors for crop protection. Further characterization of the CC-like NLRs, which have been lost in angiosperms, could provide valuable insights into plant immune signalling and the evolution of disease resistance.

## Methods

### Fern and microbe material used in this study

A collection of fern species and filamentous microbial isolates (Additional file1:Table S1) was assembled for a systematic evaluation of cross-compatibility. Some fern specimens were obtained from external laboratories, others were collected from the wild, and some were purchased from commercial sources. Most ferns used here belong to the *Polypodiales* order, the largest order of ferns. Additionally, three species from the *Equisetales* order, the sister lineage to all other ferns, were included (Additional file2:Fig.S1). Only *Pteris vittata* could be successfully maintained and propagated under our laboratory conditions, while commercial ferns did not survive long-term. However, we still have access to the ferns harvested from the wild, due to their known geographic sampling area (Castanet-Tolosan, FR).

For microbial isolates, some were isolated from the bryophyte *Marchantia polymorpha* [36] while others belong to our institutional collection (Additional file1:Table S1 and Additional file2:Fig.S1). These include broad-spectrum plant pathogens such as the ascomycete fungi *Sclerotinia sclerotiorum* [122]; *Colletotrichum spp.* (many of them were kindly provided by Dr Richard O’Connell, BIOGER, INRAE, Versailles-Saclay, France) [17]; or *Fusarium spp.* [18, 35], as well as host-specific pathogens like the oomycete *Aphanomyces euteiches* (specialist of Fabaceae) [123], and fungi not commonly considered pathogenic, such as *Biscogniauxia mediterranea*, *Coniochaeta sp.,* and *Hypoxylon sp.* [36].

### Plant growth conditions

Ferns at the sporophytic stage were grown in pots containing compost and pozzolan (3:1) in a greenhouse supplemented with LED lightning, maintaining a 16-h light/8-h dark photoperiod. Temperature was set at 22 °C and relative humidity at 70%. Gametophytes were grown from surface-sterilized spores. Spores were sterilized as follows: 2 h imbibition in water, 10 min in 1% bleach, with 0.01% Tween-20, followed by three washes with sterile water. Spores were then sown on LS ½ medium [2.36 g/l (Caisson, LSP03), 0.8 g/l agar] in Petri dishes. Cultures were sealed with parafilm and maintained in a Snijder growth cabinet (type EB1) under 15-h light/9-h dark photoperiod at 25 °C.

### Microbe culture and inoculation

Filamentous microbes were propagated on Potato Dextrose Agar (PDA, 39 g/l) at 22 °C in the dark. For long-term storage, spores were kept in 30% glycerol at − 80 °C. For mid-term storage, mycelium was stored in the dark at 10 °C on PDA Petri dishes. For inoculation, plugs of ~5 mm × 5 mm containing actively growing mycelium were excised from 10-day-old cultures. For sporophytes, detached fronds (or stems for *Equisetum* species) were placed on a moist paper towel. Gametophytes were inoculated directly on their growth medium, approximatively 2 months after sowing, prior to sporophyte formation. PDA plugs with mycelium were placed in direct contact with the frond or gametophyte surface, and the Petri dishes were sealed with parafilm to maintain high humidity. All collected ferns were inoculated in several trials with multiple pathogens. Disease symptoms were assessed at 8 days post-inoculation (dpi) by measuring the area of brown tissue surrounding the inoculation site, using ImageJ software.

### Microscopy

For scanning electron microscopy (SEM), detached fronds or gametophytes were inoculated with *F. proliferatum*, *F. oxysporum*, or *C. nymphaeae*, using 10 µl of a solution adjusted to 10^5^ spores/ml. Samples were collected at different infection stages for microscopic observations: spore germination (2 dpi) and mycelium colonization (6–17 dpi). 1 × 1 cm sections of fronds were fixed in a solution of 0.05 M sodium cacodylate buffer (pH 7.2) containing 2.5% glutaraldehyde, dehydrated through an ethanol series, and critical-point dried using liquid CO₂. Samples were mounted on the observation plate using conductive silver paint and sputter-coated with platinum. Images were acquired using a Quanta 250 FEG FEI scanning electron microscope at 5 kV, with a spot size of 3 and a working distance of 10 mm. For florescence microscopy with *Colletotrichum nymphaeae*, detached fronds were harvested at 13 dpi after inoculation using 10 µl of a solution adjusted to 10^5^ spores/ml. Cross Sects. (100 µm thick) were obtained using a Leica VT1000S vibratome and incubated for 15 min in 1X phosphate buffer saline (PBS) with 0.01% calcofluor and 5 mg/l of Wheat Germ Agglutinin-AlexaFluor-488 (WGA-AF488). For fluorescence microscopy with *Fusarium oxysporum*, detached fronds were harvested at 4 dpi after inoculation using agar plugs containing actively growing fungus. Cross Sects. (100 µm thick) were obtained using a Leica VT1000S vibratome and incubated for 15 min in 1X phosphate buffer saline (PBS) with 1 mg/l of Wheat Germ Agglutinin-AlexaFluor-488 (WGA-AF488). Cross sections were imaged using a Nikon Eclipse TI inverted Wide-field microscope with a color CMOS camera DS Ri2 and driven by Nikon NIS software. We used 10x (N.A 0.3) or X20 (N.A 0.45) objectives. Fluorescence of WGA-AF488, to stain the fungal cell wall, was observed through a bandpass GFP B filter set (ex, 475/50 nm; dich, 505 nm; em, 535/50 nm). Autofluorescence of chloroplasts was observed with a longpass B2-A filter set (ex, 470/50 nm; dich, 505 nm; em, 520 nm). Fluorescence of calcofluor, to stain cellulose of the plant cell wall, was observed by a bandpass DAPI filter set (ex, 377/50 nm; dich, 409 nm; em, 447/60 nm).

### NLR and RLK/RLP mining

Immune receptors were predicted across available fern genomes and representative species from other land plant lineages (Additional file1:Table S1). To identify putative cell-surface immune receptors, we extracted RLKs and RLPs using RGAugury v2.2 [124] which were further classified based on domain composition (Additional file1: Table S2). For intracellular immune receptors, NLRs (Additionalfile1: Table S3) were identified using both RGAugury and NLRtracker v0fe62b3 [63, 124], followed by functional domain annotation with HMMER v3.2.1 to confirm the presence of NB-ARC domains. We assigned each NLR to a category based on NLRtracker classification and/or identification of typical domains by HMM (e.g., TIR, NB, and LRR for TIR-NLR). NLRs that could not be categorised using this method were labelled as “UNDETERMINED.” A subset of 3,775 NLRs (from 3637 proteins) was retained for phylogenetic analysis. Protein sequences were aligned using MUSCLE v5.1.0 and trimmed with trimAl v1.4.1 to remove poorly aligned positions. Maximum-likelihood phylogenies were reconstructed using IQ-TREE v2.2.2.6, with the best-fitting evolutionary model selected via ModelFinder and branch support assessed with 10,000 replicates of both sh-aLRT and ultrafast bootstrap. We used the NB-ARC domain only, as it is the most conserved domain of NLRs, while the LRR domain is under diversifying selection [106]. The resulting phylogeny was visualized and annotated with iTOL v7. NLRs were classified into functional groups based on domain composition. For cell-surface receptors, RLK/RLPs were identified using RGAugury, followed by functional domain annotation with HMMER v3.2.1 to assess the nature of the ectodomain. They were classified based on both RGAugury annotation and presence of typical ectodomain identified by HMM (e.g., RLK and LRR for LRR-RLK). Proteins that could not be categorised using this method were classified as “other.” Additional methodological details are provided in Additional file3:Supplemental Methods, and 10.5281/zenodo.17067744) [63, 124–129].

## Supplementary Information

Additional file 1: Supplemental tables: Table S1: List of microbes, fern and control plant species genomes used in this study; Table S2: List of RLKs used in this study; Table S3: List of NLRs used in this study; Table S4: Presence/absence of known immune genes.

Additional file 2: Supplemental figures: Fig. S1: Phylogeny of ferns and filamentous microbes tested in this study; Fig. S2: Wordclouds of unclassified fern RLK/RLP; Fig. S3: Predicted structures of NLR N-terminal domains.

Additional file 3: Supplemental Method: Detailed Methods for NLR and RLK/RLP Mining.

## Data Availability

All novel data and datasets presented in this study are available as main or supporting information.

## Abbreviations

AF: AlexaFluor
AMP: Antimicrobial peptide
ANOVA: Analysis of variance
ARM: Armadillo repeat motif
CC: Coiled-coil
CNL: CC-type nucleotide-binding leucine-rich repeat (NLR)
CRR: Cysteine-rich receptor
CSP: Common symbiosis pathway
dpi: Days post-inoculation
DAPI: 4′,6-Diamidino-2-phenylindole
DAMP: Damage-associated molecular pattern
DUF: Domain of unknown function
EDVID: Conserved motif in CC-NLRs (specific to asterids/monocots)
EP: EDS1/PAD4/SAG101 complex
ETI: Effector-triggered immunity
GFP: Green fluorescent protein
HMM: Hidden Markov Model
HMMER: Hidden Markov Model-based sequence alignment tool
ICS1: Isochorismate synthase 1
iTOL: Interactive Tree of Life
LRR: Leucine-rich repeat
LS ½: Half-strength Linsmaier and Skoog medium
MB: Megabase pairs
NLR: Nucleotide-binding domain and leucine-rich repeat receptor
NB-ARC: Nucleotide-binding domain shared among APAF-1, R proteins and CED-4
NRC: NLR required for cell death
ORF: Open reading frame
PAD4: Phytoalexin deficient 4
PBS: Phosphate buffered saline
PDA: Potato dextrose agar
pTM: Predicted template modelling score
PTI: PAMP-triggered immunity
PAMP: Pathogen-associated molecular pattern
RGA: Resistance gene analogs
RLK: Receptor-like kinase
RLP: Receptor-like protein
RPW8: Resistance to powdery mildew 8
SAR: Systemic acquired resistance
SA: Salicylic acid
SEM: Scanning electron microscopy
Sh-aLRT: Shimodaira–Hasegawa approximate likelihood ratio test
SARD1: SAR deficient 1
SNP: Single nucleotide polymorphism
TIR: Toll/interleukin-1 receptor domain
TNL: TIR-type NLR
TPR: Tetratricopeptide repeat
WAK: Wall-associated kinase
WGA-AF: Wheat germ agglutinin conjugated with AlexaFluor
X: Variable (used in NLR nomenclature)
