# Supplementary material for: Exploring fern pathosystems and immune receptors to bridge gaps in plant immunity
Source: BMC Biol. 2025 Oct 9;23:301. doi: 10.1186/s12915-025-02413-6 (PMC12512272; doi:10.1186/s12915-025-02413-6)
Supplement: Supplementary file 2 — Additional file 2: Supplemental figures: Fig. S1: Phylogeny of ferns and filamentous microbes tested in this study; Fig. S2: Wordclouds of unclassified fern RLK/RLP; Fig. S3: Predicted structures of NLR N-terminal domains. [file 12915_2025_2413_MOESM2_ESM.docx]

# Supplemental Figures


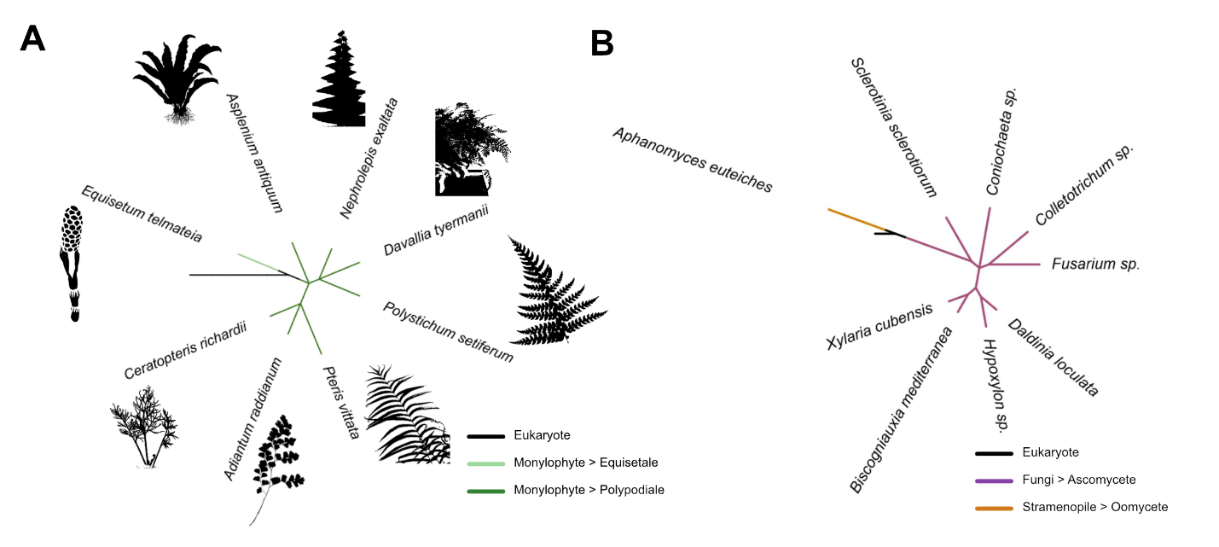


**Supplemental Figure 1: Phylogeny of ferns and filamentous microbes tested in this study**

**A.** Phylogenetic tree of the fern species tested, generated using ‘phyloT’ (<https://phylot.biobyte.de/>) and visualised with iTOL (<https://itol.embl.de/>). The black branch represents eukaryotes, the light green corresponds to Equisetales and dark green to Polypodiales. Cartoons sources: *A. antiquum*, *A. raddianum*, *E. telmateia* (phylopics, <https://www.phylopic.org>), *C. richardii* (modified from <https://c-fern.org/>), *N. exalatata*, *P. setiferum*, *P. vittata* (this study). **B.** Phylogenetic tree of the microbial species tested, generated using ‘phyloT (<https://phylot.biobyte.de/>) and visualised using iTOL (<https://itol.embl.de/>). The black branch indicates eukaryotes, purple indicates opistokonts, orange represents stramenopiles.


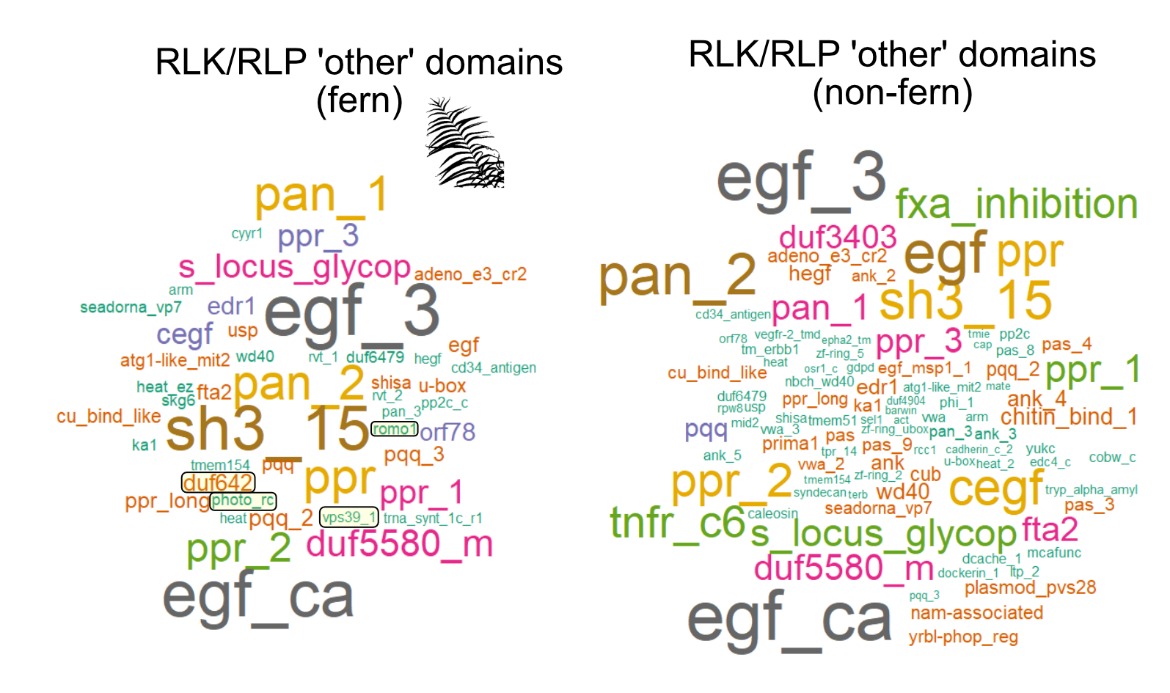


**Supplemental Figure 2: Wordclouds of fern RLK/RLP ‘others’ and disN-NLR predicted protein domains**

Wordcloud representation of HMM domains detected in the ‘other’ category of RLKs/RLPs (mostly RLKs). Kinases and common signalling domains (such as EF-hand) were excluded for clarity. HMM domains unique to ferns are highlighted in black squares (romo1, vps39, mcm, photo_rc and duf642). Figure generated with the R package ‘wordcloud’.


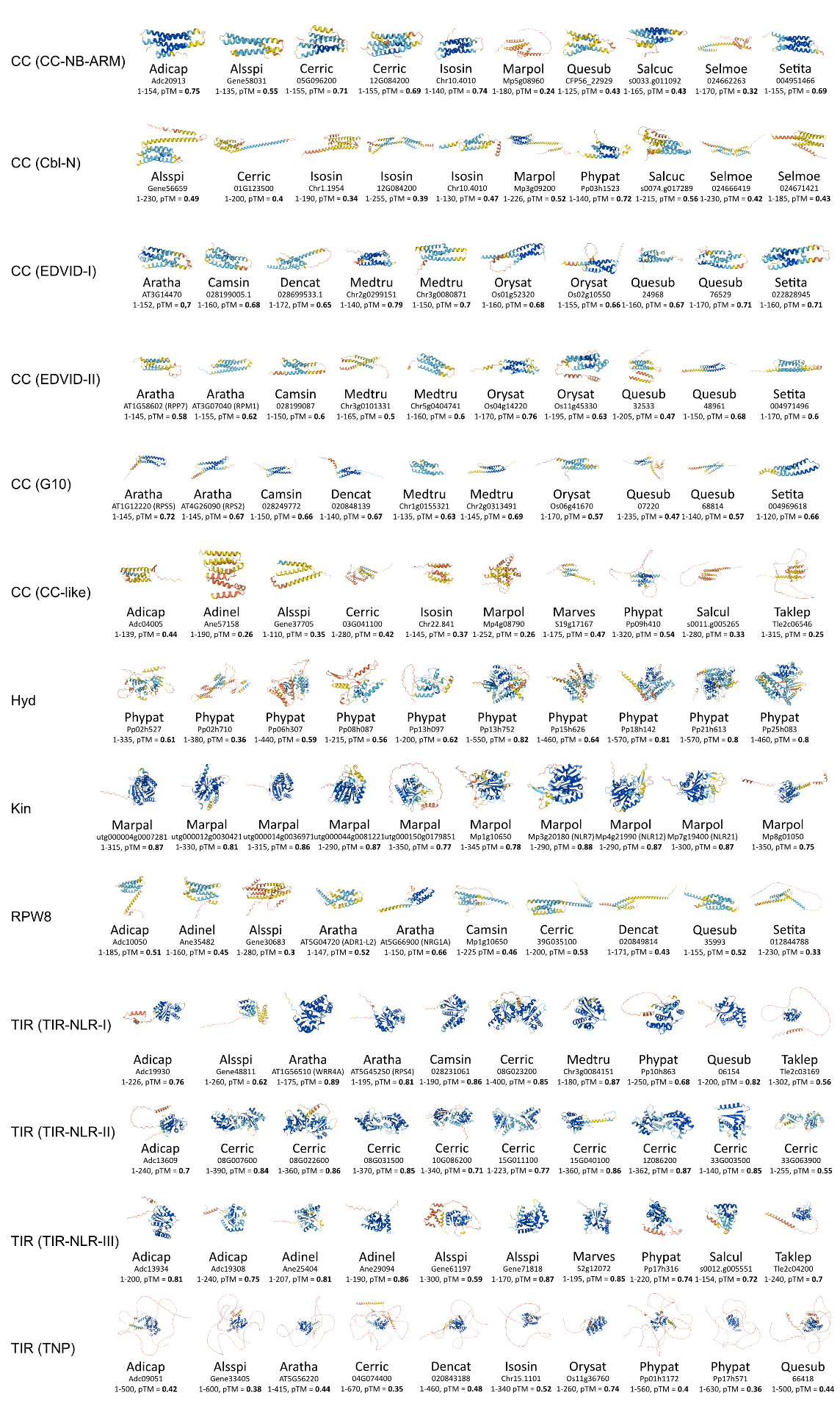


**Supplemental Figure 3:** **Predicted structures of NLR N-terminal domains**

This figure represents the N-terminal domain (from start to NB-ARC, as predicted by HMM) of 10 NLRs from the clades presented in the phylogeny on Figure 5A and Table S3. Structures were predicted by AlphaFold (<https://alphafoldserver.com/>). Under each structure, is indicated the plant species (see Table S1 for description), the gene name, selected amino acids and the AlphaFold confidence score (pTM). Orange indicates a very low confidence, yellow a low confidence, light blue a good confidence and dark blue a very high confidence.
